# Bilateral lymphadenopathies on mammograms: a case of mixed connective tissue disease and psoriatic arthropathy

**DOI:** 10.1259/bjrcr.20220077

**Published:** 2023-01-23

**Authors:** Emilia Giambersio, Veronica Magni, Francesco Sardanelli

**Affiliations:** 1 Postgraduate School in Radiodiagnostics, Università degli Studi di Milano, Milano, Italy; 2 Department of Biomedical Sciences for Health, Università degli Studi di Milano, Milano, Italy; 3 Unit of Radiology, IRCCS Policlinico San Donato, San Donato Milanese, Italy

## Abstract

Axillary lymphadenopathy is defined as abnormality (*e.g.,* increase in size or density) of lymph nodes in the armpit, caused by malignant diseases such as metastases (mainly from primary breast cancer), lymphoma, or leukaemia as well as benign conditions such as infectious or autoimmune systemic diseases. Appropriate imaging and pathological examinations on needle samples, together with accurate clinical correlation are needed for a correct diagnosis and management.

Herein, we report a case of a 47-year-old female presented at our department of radiology for her annual mammographic screening. Mammography demonstrated multiple bilateral, enlarged, although benign-appearing axillary lymph nodes. While both breasts showed no sign of malignancy on mammograms, the lymphadenopathies suggested a potential underlying inflammatory process. Previous mammography performed five years before did not present any lymphadenopathy. The patient, recalled for additional breast and axillary ultrasound and for clinical correlation, reported that she had been suffering for at least four years from an autoimmune systemic disease, mixed connective tissue disease, recently overlapping with psoriatic arthropathy, thus explaining the aetiology of reactive lymph nodes enlargement.

## Clinical presentation

**Figure 1.**
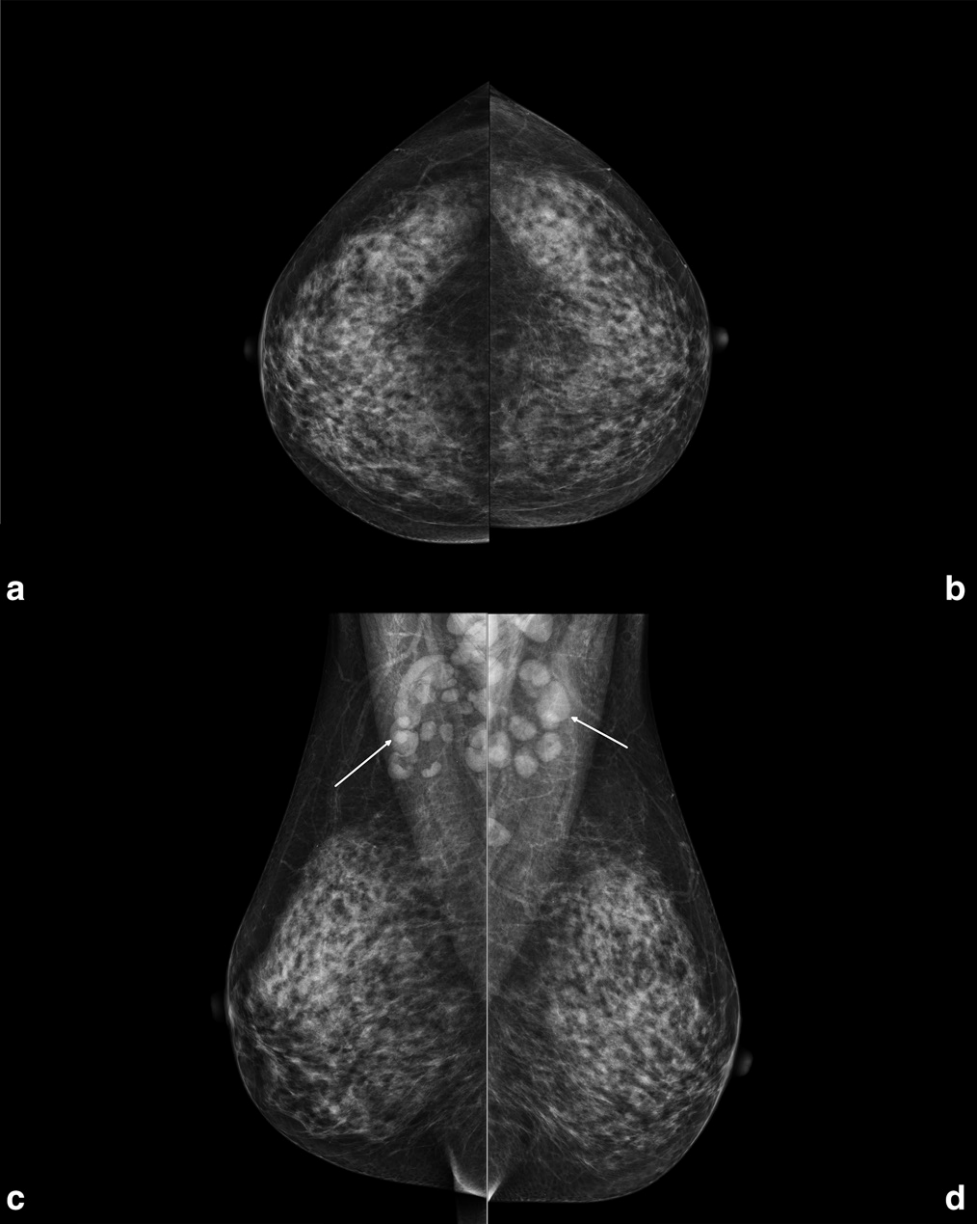
Right and left craniocaudal (A, **B**) and mediolateral oblique (C, **D**) views performed in January 2022 in a 47-year-old female attending her regular screening mammography. In the mediolateral oblique views multiple enlarged benign-appearing axillary lymph nodes are visible bilaterally (white arrows), at least 16 on the left side (the largest one of 18 mm) and at least 11 on the right side (the largest one of 33 mm). In both projections no findings suspicious for breast cancer were found.

**Figure 2.**
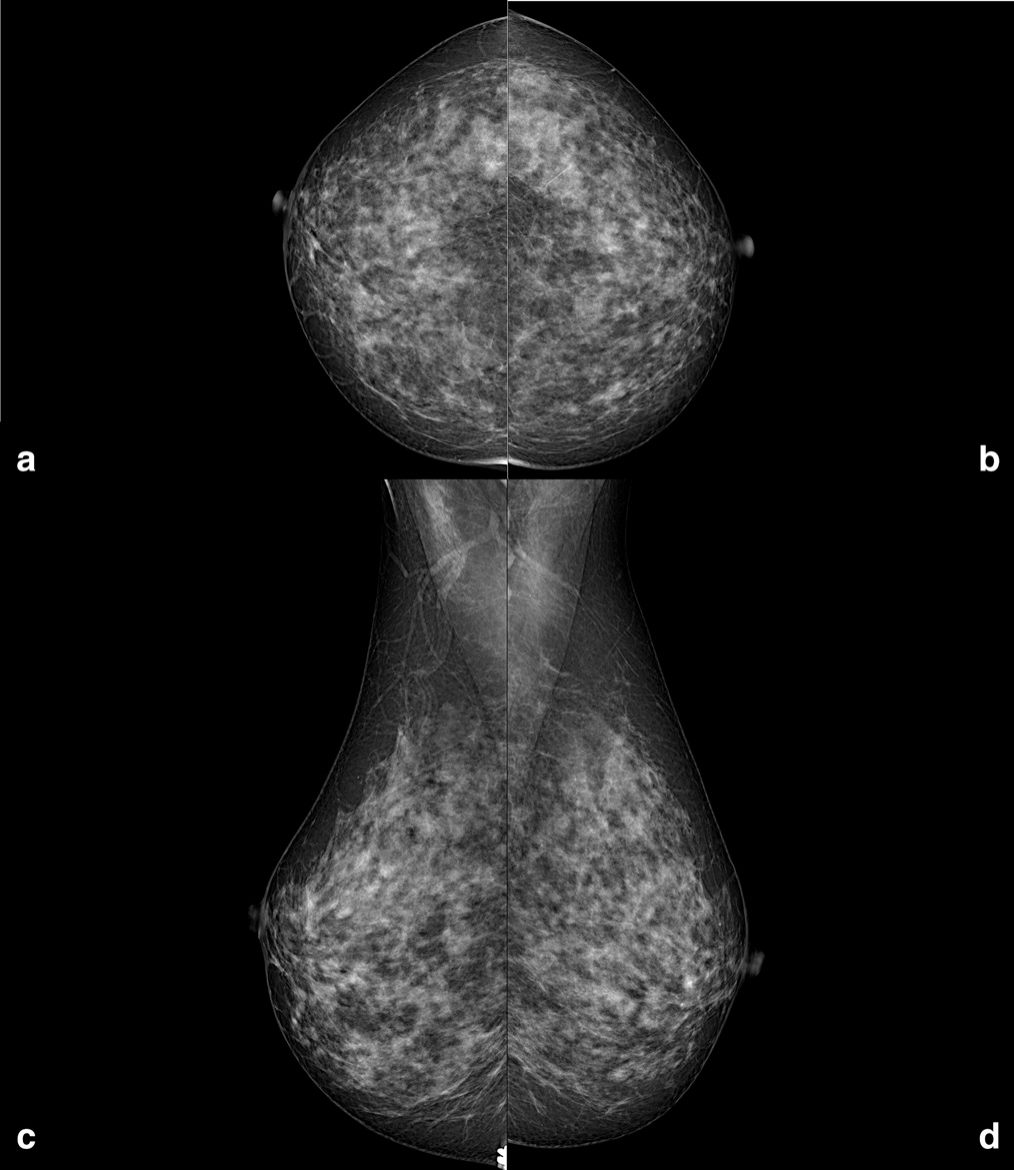
Right and left craniocaudal (**A, B**) and mediolateral oblique (**C, D**) mammographic views performed in July 2017. Neither enlarged axillary lymph nodes, nor breast suspicious findings are visible.

In the population-based organised breast cancer screening programme provided by our local Agency for Health Protection, biennial mammography is offered to females aged 50–74 years, while annual mammography is offered to females aged 45–49 years.^1^ A 47-year-old female presented at our department of radiology for her annual mammographic screening. Mammography demonstrated many enlarged axillary lymph nodes bilaterally (at least 16 on the left side, the largest one of 18 mm; at least 11 on the right side, the largest one of 33 mm), while not showing any breast finding suspicious for cancer on both sides (Figure 1). Of note, the patient reported neither breast or axillary symptoms, nor recent vaccinations. Importantly, the patient did not present any axillary adenopathy in the previous mammographic screening round, in 2017 (Figure 2).

## Differential diagnosis

In this patient, the mammographic features of the enlarged lymph nodes were not suspicious for malignancy because of their oval shape, visible fatty hilum, and bilateralism, hinting at an inflammatory underlying process. Thus, the first differential diagnosis hypothesised was physical or chemical cutaneous injuries, like shaving or the use of aggressive skin products.^2^ However, vaccination-induced axillary lymphadenopathy had also to be considered as a potential differential diagnosis, as seen during the ongoing COVID-19 vaccination campaign.^3^ Thus, recent vaccinations data such as dose, date, and side of injection was investigated. Other possible differential diagnoses were malignant diseases, such as lymphoma, leukaemia, melanoma metastases, etc. (even if not consistent with the presentation of these enlarged lymph nodes) and also infectious diseases such as tuberculosis, cat-scratch disease, human immunodeficiency virus, mononucleosis, cytomegalovirus, toxoplasmosis, and brucellosis.^4^ Bilateral axillary lymphadenopathy could also be associated to systemic connective tissue diseases such as rheumatoid arthritis, lupus erythematosus, psoriatic arthropathy, and scleroderma.^5^ Finally, a rare condition that should be taken into consideration is bilateral breast cancer, which can be either metachronous (detected after 3–6 months from the initial diagnosis) or synchronous (detected within 3–6 months from the initial diagnosis) and accounts for around 1–3% of all breast cancer diagnoses.^6–8^

## Investigations and imaging findings

**Figure 3.**
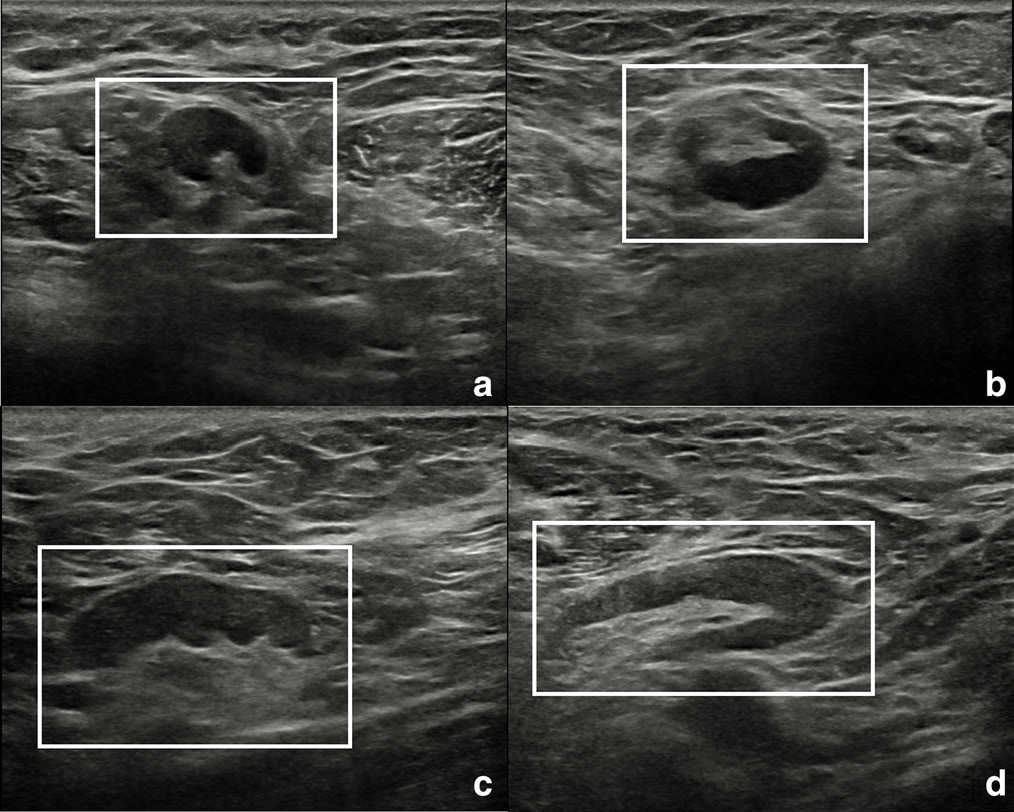
Ultrasound scans of right (**A, B**) and left (**C, D**) armpits showing lymph nodes with short to long axes ratio <0.5 (white rectangles). Oval shape, bilateralism, and visible fatty hilum are suggestive for a benign, reactive aetiology.

**Figure 4.**
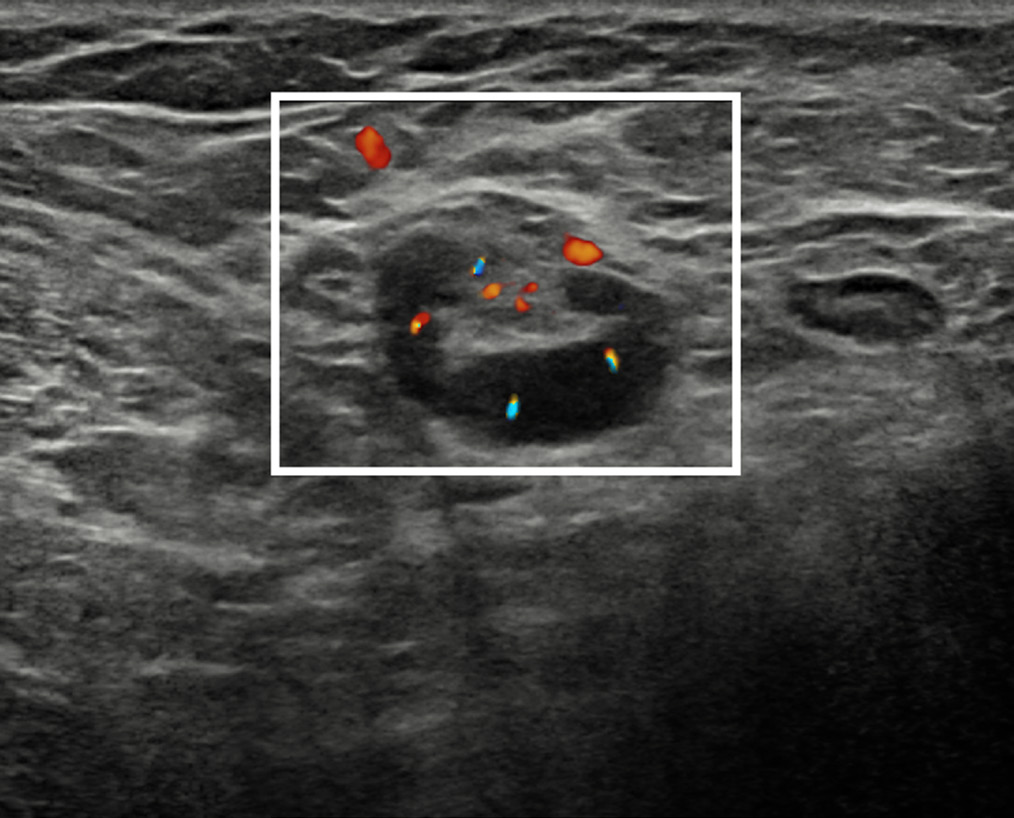
Ultrasound scan with Doppler signal overlapped on B-mode images of the right armpit showing an enlarged lymph node with increased intra- and extranodal vascularization and congested hilum (white rectangle), suggestive for active inflammation.

The patient was recalled to perform further assessment of the breast and of the armpits, and to collect a more detailed clinical history. Additional bilateral breast and axillary ultrasound was performed (Figure 3), confirming the presence of numerous enlarged lymph nodes, the largest one measuring 30 × 5 mm, with Doppler signal showing intra- and extranodal vascularization, playing in favour of an active inflammatory process (Figure 4). Furthermore, the patient reported that such findings were already visible at a previous chest computed tomography, performed in November 2021 (images not available). More importantly, she reported that she had been diagnosed with mixed connective tissue disease in 2018 overlapping with psoriatic arthropathy since 2020. Hence, the inflammatory nature of the axillary lymphadenopathies was confirmed, and the female was advised to continue the regularly scheduled mammographic screening.

## Discussion

Mixed connective tissue disease was first described as a new autoimmune rheumatic disease in 1972 based on the claim of a distinct clinical picture associated with antiribonucleoprotein antibody positivity. This systemic syndrome consists of overlapping features between scleroderma, systemic lupus erythematosus, polymyositis/dermatomyositis, with a favourable prognosis.^9^ Although breast involvement in autoimmune diseases is uncommon, possible presentation at mammography includes either localised disease or indirect signs of systemic involvement. Imaging findings can include breast masses, axillary lymphadenopathies, calcifications, and skin changes.^10^

The interest of this case lies in the fact that diffuse lymphadenopathies detected at screening mammography can lead to multiple differential diagnoses and are not always synonymous with lymph node malignancy, including metastases from overt or occult breast cancer. According to the American College of Radiology Breast Imaging Reporting and Data System (ACR BI-RADS) enlarged axillary lymph nodes require clinical correlation and additional imaging, particularly when new or modified in comparison to previous examinations.^11^ Thus, the patient’s medical history may explain the axillary lymphadenopathy, avoiding further evaluation.

Axillary ultrasound is the main imaging tool for the assessment of lymph nodes, which are divided into three levels in relation with pectoralis minor muscle: level I axillary lymph nodes are lateral to the lateral border of the pectoralis minor muscle; level II axillary lymph nodes are between the medial and lateral borders of the pectoralis minor muscle; level III axillary lymph nodes are medial to the medial margin of the pectoralis minor muscle and inferior to the clavicle.^12^

Infectious diseases, lymphoma, leukaemia, breast cancer, and melanoma of the areas drained by axillary lymph nodes (*i.e*., upper arms, trunk, and breast) must always be considered as possible differential diagnoses. In particular, axillary lymph nodes have been reported to be involved in the 11.9% of cases of Non-Hodgkin and Hodgkin lymphomas.^13^ Rarely, axillary lymphadenopathy (mostly unilateral) can also originate from a primary breast lymphoma, with regional nodal involvement at diagnosis ranging from 28.3 to 37.9%.^14–16^ It is also common for patients suffering from connective tissue diseases such as rheumatoid arthritis, systemic lupus erythematosus, etc. to present bilateral axillary lymphadenopathies, which reflect the inflammatory activity and severity of the disease, and can be visualised on mediolateral oblique mammographic projections.^5^

In all these cases, the appearance and morphology of lymph nodes observed on mammograms, and subsequently evaluated with bilateral axillary ultrasound, can be suggestive of their malignancy or rather of their benign nature: according to the ACR BI-RADS, even although there is no consensus and there is wide individual variability, a normal axillary lymph node may be up to 2 cm in its longest dimension and contain hyperechoic fatty hilum.^17^ Indeed, while fatty hilum, oval shape, and regular margins are characteristic features of normal or benign lymph nodes, round shape, absence of the fatty hilum, and increased eccentric or focal cortical thickness are characteristics concerning for malignancy.^18^ Of note, the cortical thickness measure used as a cut-off point for metastatic disease is highly variable in literature, ranging from 2.3 to 3 mm.^19,20^ Hence, the cortex to hilum ratio can also be adopted, suggesting malignancy when the former parameter is greater than or equal to the latter.^21^ Furthermore, bilateralism and the enlargement of many lymph nodes in a specific chain are also very important, suggesting a systemic aetiology and potentially excluding the involvement of singular nodes, as is in the case of metastatic diseases.

Nonetheless, comparison with previous imaging examinations and correlation with the clinical status of the patient are important, especially when lymphadenopathies are of new identification, like in our case.^5^ Thus, investigation and collection of a detailed anamnesis have a key role in the clinical decision-making.

It is also important to recall the possibility of occult breast cancer, described as an axillary metastatic carcinoma with no evidence of a primary breast lesion, accounting for up to 1% of all breast cancers. In such cases breast MRI or contrast-enhanced mammography should be performed, when also digital breast tomosynthesis and ultrasound are negative.^22^

Finally, lymph node biopsy is recommended in any case of doubt, when additional breast imaging examinations yield to inconclusive results, when the lymph nodes do not show clearly benign features, and when the clinical history of the patient is unknown or not reassuring.

## Learning points

- Dealing with axillary lymphadenopathies, several differential diagnoses must be taken into consideration, including benign and malignant diseases.
- Implementation of clinical information and imaging examinations is pivotal for the decision-making process in the management of axillary lymphadenopathy.
- Exclusion of primary breast cancer and morphologic characterisation of lymph nodes using mammographic views and additional imaging (*i.e.,* digital breast tomosynthesis, ultrasound, additional views, breast MRI) might guide the diagnosis.
- In case of bilateral axillary lymphadenopathy, autoimmune systemic conditions such as mixed connective tissue disease must be considered as the potential underlying condition.
